# Engineering the Future of Dental Health: Exploring Molecular Advancements in Dental Pulp Regeneration

**DOI:** 10.3390/ijms241411453

**Published:** 2023-07-14

**Authors:** Matthias Widbiller, Kerstin M. Galler

**Affiliations:** 1 Department of Conservative Dentistry and Periodontology, University Hospital Regensburg, Franz-Josef-Strauß-Allee 11, D-93093 Regensburg, Germany; 2 Department of Operative Dentistry and Periodontology, Friedrich-Alexander-University Erlangen-Nuernberg, D-91054 Erlangen, Germany; kerstin.galler@uk-erlangen.de

Protected by the surrounding mineralized barriers of enamel, dentin, and cementum, dental pulp is a functionally versatile tissue that fulfills multiple roles. In addition to the perception of thermal and mechanical stimuli as a warning system and the deposition of dentin, the pulp performs a variety of immunological functions against invading microorganisms, both in terms of recognition and defense. Especially, in young patients, dental pulp is essential for the completion of root development, and early pulp necrosis results in fracture-prone teeth with fragile root walls [1,2,3,4]. Whether in young or adult patients, the loss of pulp tissue due to caries or trauma requires a therapeutic intervention by means of root canal treatment and obturation with a synthetic material.

In recent years, innovative attempts have been made to regenerate dental pulp using advanced molecular biology techniques [5,6]. Promising approaches, based on tissue engineering and regenerative medicine, have been developed for this purpose [7,8]. In this context, stem cell-based or primarily cell-free approaches use specifically tailored scaffold materials and signaling molecules to achieve pulp regeneration, both in terms of tissue microarchitecture and functionality (Figure 1). Several of these approaches already take into account the requirements of potential clinical applications [9,10,11].

Essentially, all these methods follow the traditional principles of tissue engineering. They involve the incorporation of stem cells and growth factors into a scaffold material (Figure 2). Ongoing scientific research is, therefore, focusing on advances in three key areas: stem cell biology, scaffold materials, and molecular signaling. The ultimate goal is to bring us closer to the potential of biological regeneration within the root canal. Consequently, the purpose of this Special Issue is to present scientific advances in pulp regeneration, which play a crucial role in translating knowledge from the research setting to the clinic.

Accurately defining the goal of regenerative endodontic treatment is paramount [7,15,16,17]. Biologically, this treatment aims to fully restore all cellular and structural elements of the dentin–pulp complex, while at the same time enabling the newly formed tissue to perform all functions of the original pulp tissue. As it is difficult to assess these criteria directly in a clinical setting, scientists are more dependent than ever on appropriate experimental models to gain insights into treatment outcomes at the tissue and cell level.

Ohlsson et al. have undertaken a comprehensive compilation of preclinical study models [18]. Their review provides an overview of various ectopic, semi-orthotopic, and orthotopic in vitro and in vivo models to investigate regenerative endodontics. One key focus is the critical evaluation of the monolayer and three-dimensional cell cultures. Additionally, a semi-orthotopic transplantation model, as well as various animal models for orthotopic in-vivo-investigations, are presented and critically contrasted against each other. Nowadays, different three-dimensional cell culture techniques offer viable alternatives to animal experimentation. Certain research questions can be easily addressed in vitro, and the continued development of organoid and spheroid cultures, for example, may expand their applications in the future. However, in order to gain further insight into the results in a physiological environment, animal studies are still necessary, and the final evaluation of the research objective must be carried out using an in situ approach. Considering the diversity of in vitro and in vivo study models available, there is no single model that can answer all questions related to pulp regeneration. Depending on the research question, the appropriate model situation must be selected.

To provide an overview of the possible outcomes of regenerative endodontic procedures and to highlight the inconsistency of criteria for the successful regeneration in both animal models and human studies, Minic et al. conducted a systematic review [19]. The aim was to categorize the characteristics of the resulting tissues that could be evaluated in each study design. In addition to the tissue engineering approaches, the search also included blood clot-based procedures. With the latter, tissue formation is initiated by induced bleeding into the root canal. The search identified 75 studies in humans and 49 studies, which reported data from animal models. In humans, the assessment criteria were mainly based on clinical and radiographic examinations, with the majority of studies reporting successful clinical outcomes with relief of symptoms, healing of apical lesions, and increases in root thickness and length. In animal studies, evaluations included both histological and radiological criteria. None of the studies included in the analysis reported successful regeneration of the dentin–pulp complex based on biological criteria. Instead, both preclinical and clinical studies demonstrated the presence of connective tissue, accompanied by cementum or bone-like tissue within the root canal. Several animal studies reported the presence of vascularized and innervated regenerated pulp, however, the clinical response to these findings remained unclear. In human studies, a proportion of patients have regained sensitivity following regenerative endodontic therapy approaches. While the formation of repair tissue may meet the clinical needs of patients and clinicians, further research is needed to identify procedures that come closer to the goal of pulp regeneration.

One key is to identify the molecular–biological signaling pathways that may drive the differentiation of individual cell types. The most distinctive cell type found in dental pulp tissue is the odontoblast, which forms a closed cell layer at the interface of dentin. This unique histological feature is of great importance, as only native odontoblasts have the ability to produce new tubular dentin, known as reactionary dentin. However, other cell types predominantly deposit hard tissue without characteristic tubular morphology. Due to the complexity of regenerating the dentin–pulp complex, many studies have focused on identifying novel molecular biological strategies. One such strategy is to target Wnt proteins, as they have demonstrated their potential as highly relevant stem cell factors capable of regulating the self-renewal and proliferation of various adult stem cell populations. This ability makes Wnt proteins suitable for maintaining homeostasis, promoting tissue healing, and facilitating regeneration. With this in mind, Florimond et al. conducted a systematic review to outline different agents that target Wnt signaling [20]. The aim was to identify strategies to harness Wnt signaling via modulatory molecules and to evaluate their applicability in the dental environment. Numerous studies have highlighted the importance of Wnt signals in the formation and repair of the dentin–pulp complex. Furthermore, research groups have successfully demonstrated that a Wnt stimulus can induce tissue regeneration. As a result, small molecule drugs that stimulate the Wnt/β-catenin pathway have emerged as a promising therapy. However, it is crucial to consider the local application of these drugs to avoid potential systemic side effects. Although there is still a need for proof-of-concept, these advancements bring us closer to realizing the potential of regenerating pulp tissue.

Cell differentiation and the development of new pulp tissue rely on stem cells, which have a high potential for specialization [21,22]. These stem cells can either be transplanted into the root canal or harvested from local tissues [23,24,25]. This leads to two concepts in endodontic tissue engineering: cell transplantation (cell-based) and cell homing (primarily cell-free). In cell-based methods, cells are cultured ex vivo and then introduced into the root canal using scaffolds containing signaling molecules. Clinical translation of this approach poses significant challenges, as it requires donor tissue or cell banks. In contrast, cell-homing methods utilize naturally residing stem or progenitor cells within the body that are locally available, bypassing any ex vivo manipulation. Here, scaffolding materials that are primarily cell-free are placed in the root canal, along with signaling molecules to attract cells from the remaining pulp tissue or the surrounding periapical space.

Among various types of stem cells associated with teeth, dental pulp stem cells (DPSCs) and apical papilla stem cells (SCAPs) are particularly suitable for cell harvesting because they are naturally found in the root canal or apical papilla at the root tip. Therefore, Smeda et al. investigated whether both types of stem cells are equally suitable for regenerative endodontic protocols [26]. To investigate this, DPSCs and SCAPs were isolated from the same donor, and their characteristics were extensively studied, with a focus on gene expression profiling during induced differentiation. The viability, proliferation, and migration of DPSCs and SCAPs appeared very similar. However, differences in the profile of secreted molecules were observed. Transcriptome analysis identified 13 biomarkers whose regulation was critical for the differentiation of both cell types. These findings suggest that DPSCs and SCAPs share many similarities and exhibit similar patterns of differentiation. From a molecular biology point of view, both types of stem cells appear to be equally suitable for dental pulp tissue engineering.

Furthermore, the possibility of cell transplantation has been extensively studied preclinically and clinically in recent years [27,28]. Recent in vivo studies have shown that pulp regeneration after cell transplantation appears promising [29,30,31]. For cell transplantation, it is of central interest to identify cell sources in the body that are readily accessible and capable of differentiating into odontoblasts. The approach of cell transplantation with DPSCs and other tooth-derived cell types is problematic because of the need for cell expansion to obtain sufficient numbers of cells and the need for a donor tooth. A possible cell source to overcome these obstacles could be the amnion, the innermost layer of the human placenta [32,33]. It contains amniotic epithelial cells (AECs), which retain plasticity and thus are able to differentiate into cells of all three embryonic layers [21,34].

Bucchi et al. set out to investigate the suitability of amnion-derived pluripotent stem cells as a potential cell source for pulp regeneration in vitro [34]. In addition to viability and cell attachment to dentin, the expression of genes associated with mineralization and odontogenic differentiation, mineralization and epithelial–mesenchymal transition was analyzed. The viability of AECs was significantly lower than that of DPSCs, whereas both AECs and DPSCs adhered to dentin. Regulation of genes associated with odontoblast differentiation and mineralization showed that AECs did not transition to an odontoblastic phenotype, but genes associated with epithelial–mesenchymal transition were significantly upregulated. In analogy, AECs showed low levels of calcium deposition after differentiation. Although pluripotent AECs adhered to dentin and had the ability to mineralize, they showed unfavorable proliferation behavior and did not undergo odontoblastic transition.

In addition to the use of stem cells from different regions of the body, teeth can also be a source of stem cells for use elsewhere in the body, e.g., for bone regeneration. Interestingly, Kunimatsu et al. pursued the idea of using stem cells from the pulp of primary teeth for bone regeneration [35]. The scientists focused on a very specific population of stem cells from deciduous human teeth (SHEDs), namely, the fraction expressing the cluster of differentiation (CD) 146 marker. CD146 is a stem cell surface antigen that is critical for angiogenic and osteogenic differentiation. Bone regeneration has been reported to be accelerated after transplantation of CD146-positive mesenchymal stem cells derived from deciduous dental pulp. The aim of this study was, therefore, to compare the effects of CD146 in a population of SHEDs using molecular biological methods. A CD146-positive cell population (CD146+) and a CD146-negative cell population (CD146-) were obtained by cell sorting. While cell proliferation was indifferent, mineralization and marker expression were highest in the CD146+ group. CD146+ SHEDs showed the highest osteogenic differentiation potential and, therefore, may represent a valuable cell population for bone regeneration therapy.

Turning back to tissue engineering of the dental pulp, the root canal poses a number of challenges for stem cells. One of these is bacterial infection, which sometimes cannot be completely eliminated, despite intensive disinfection that knowingly affects the outcome [36,37]. If suitable stem cells are successfully introduced into the root canal, residual bacteria may influence their differentiation and thus affect the outcome of regeneration [38,39]. To explore the potential impact of oral bacteria on regenerative endodontic treatment, Razghonova et al. conducted a study, focusing on the interaction between stem cells from the apical papilla and these bacteria [40]. Using RNA-seq transcriptomic analysis, the researchers investigated the impact of Enterococcus faecalis and Fusobacterium nucleatum, along with their metabolites, on the differentiation of SCAPs towards osteogenic and dentinogenic lineages in vitro. Gene analysis revealed that bacterial metabolites had a significant influence on SCAPs, and the transcriptome profiles indicated a negative effect on the osteogenic and dentinogenic differentiation when exposed to E. faecalis or F. nucleatum. The findings demonstrated that F. nucleatum, E. faecalis, and their metabolites can alter the expression of genes involved in dentinogenesis and osteogenesis, potentially affecting the outcomes of regenerative endodontic procedures.

While disinfection is crucial, the long-term challenge lies in addressing microorganisms that may evade disinfection [39]. Therefore, the development of scaffold materials with antimicrobial properties is highly desirable for applications in dental pulp tissue engineering. In this context, Ayoub et al. utilized electrospinning techniques to create a gelatin methacryloyl (GelMA) fiber loaded with azithromycin (AZ) [41]. Gelatine itself is a natural biomaterial derived from hydrolyzed collagen, containing an alternating sequence of the arginine, glycine, and aspartate (RGD) tripeptide motif, which promotes cell adhesion and very low exhibition of immune responses. The study encompassed a comprehensive investigation of various aspects, including the morphology and diameter of the fibers, the incorporation of AZ, the mechanical properties of the fibers, the degradation profile, and antimicrobial activity. Furthermore, in vitro compatibility with DPSCs and an in vivo evaluation of the inflammatory response were conducted. The findings demonstrated that the presence of AZ did not lead to significant toxicity. In vivo results revealed increased vascularization, mild inflammation, and the presence of mature, well oriented collagen fibers that were interwoven with the engineered fibers.

The same research group carried out a study to explore the potential of enhancing the GelMA scaffold material by combining it with other antimicrobial agents. Ribeiro et al. incorporated clindamycin or metronidazole microparticles into the scaffold [42]. The experimental hydrogels were subjected to various analyses, including swelling and degradation assessments, evaluation of toxicity towards dental stem cells, and examination of antimicrobial activity against endodontic pathogens. The introduction of antibiotic-loaded fibrous microparticles into GelMA resulted in an increase in both the swelling ratio and degradation rate of the hydrogels, and the hydrogels containing antibiotic-loaded fibrous microparticles exhibited significant antibiofilm effects, although cell viability was compromised. Overall, these results suggest that the injectable hydrogels with antibiotic-loaded fibrous microparticles have promising clinical potential for the effective eradication of endodontic infections.

There is a continuous flow of new developments and innovations in the field of scaffold materials. While antimicrobial properties would be helpful, there are many requirements to be met for pulp tissue engineering applications [43], e.g., control of the mechanical properties, such as stiffness and setting time, of the material used for preparation and injection into the root canal, is critical. Furthermore, it is important that the material allows for the binding, stabilization, and controlled release of signaling molecules. The cured material should be permeable for nutrients and metabolites, as well as biocompatible and biodegradable, to allow cell migration and transformation of the material into new tissue. Here, natural biomaterials have proven to be especially suitable [44,45]. One innovative natural material is carrageenan, a polysaccharide obtained from red algae [46]. Among them, kappa- and iota-carrageenan have received much attention as scaffold materials due to their gelling properties in the food industry, but also in regenerative medicine due to their cytocompatibility. The biological properties of kappa-carrageenan are based on its three hydroxyl groups per disaccharide repeating unit, which make it hydrophilic, as well as its one negatively charged sulphate group, which enables chemical reactions. Loukelis et al. investigated the effect of kappa-carrageenan on the behavior of dental pulp stem cells. Kappa-carrageenan/chitosan/gelatin scaffolds cross-linked with KCl were compared with carrageenan/chitosan/gelatin without KCl and chitosan/gelatin. Cell viability was significantly increased over the experimental period, as was alkaline phosphatase (ALP) activity and the expression of odontogenic marker genes. The results demonstrate that incorporation of kappa-carrageenan into scaffolds significantly enhances the odontogenic potential of DPSCs and may support dentin–pulp regeneration.

In summary, the field of dental pulp regeneration has made significant progress in recent years. Preclinical and clinical studies have provided insight into the results of regenerative endodontic procedures. Although repair tissue formation has been observed, true regeneration of the dentin–pulp complex, based on biological criteria, has yet to be achieved. Further research is needed to overcome existing challenges and bridge the gap between preclinical findings and clinical applications. Continued efforts in stem cell biology, scaffold materials, molecular signaling, and experimental models will bring us closer to the goal of true pulp regeneration and restoration of pulp tissue functionality.

## Figures and Tables

**Figure 1:**
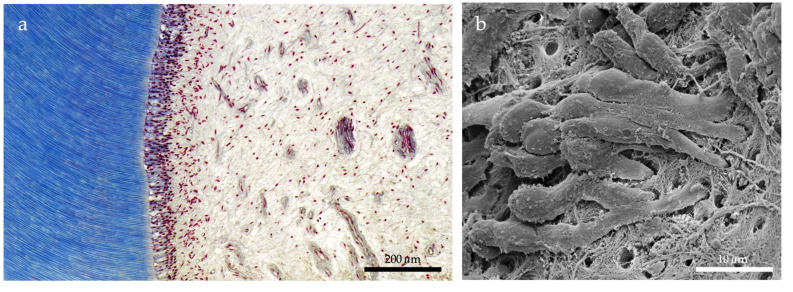
The goal of regenerative endodontics is the restoration of the dentin–pulp complex with all its cellular and structural elements. (**a**) Histological image of the dentin–pulp complex. The odontoblast layer lies on tubular dentin (blue). Vessels and nerve fibers cross the pulp tissue. (**b**) Odontoblasts seen through a scanning electron microscope. The cells extend their processes into the dentin tubules [12].

**Figure 2:**
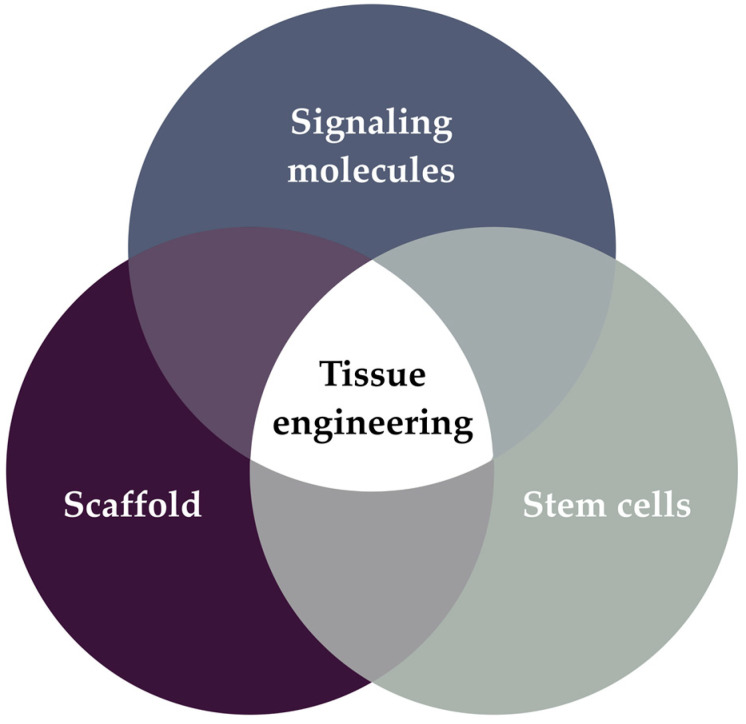
The tissue engineering paradigm involves delivering stem cells and signaling molecules in a scaffold material [13,14].
